# Impact of the temperature on the interactions between common variants of the SARS-CoV-2 receptor binding domain and the human ACE2

**DOI:** 10.1038/s41598-022-15215-5

**Published:** 2022-07-07

**Authors:** Catherine Forest-Nault, Izel Koyuturk, Jimmy Gaudreault, Alex Pelletier, Denis L’Abbé, Brian Cass, Louis Bisson, Alina Burlacu, Laurence Delafosse, Matthew Stuible, Olivier Henry, Gregory De Crescenzo, Yves Durocher

**Affiliations:** 1 Department of Chemical Engineering, Polytechnique Montreal, Montreal, QC H3T 1J4 Canada; 2 Human Health Therapeutics Research Centre, National Research Council of Canada, Montreal, QC H4P 2R2 Canada; 3 Department of Biochemistry and Molecular Medicine, University of Montreal, Montreal, QC H3T 1J4 Canada

**Keywords:** Biophysical chemistry, Glycobiology, Proteins, Biochemistry

## Abstract

Several key mutations in the Spike protein receptor binding domain (RBD) have been identified to influence its affinity for the human Angiotensin-Converting Enzyme 2 (ACE2). Here, we perform a comparative study of the ACE2 binding to the wild type (Wuhan) RBD and some of its variants: Alpha B.1.1.7, Beta B.1.351, Delta B.1.617.2, Kappa B.1.617.1, B.1.1.7 + L452R and Omicron B.1.1.529. Using a coiled-coil mediated tethering approach of ACE2 in a novel surface plasmon resonance (SPR)-based assay, we measured interactions at different temperatures. Binding experiments at 10 °C enhanced the kinetic dissimilarities between the RBD variants and allowed a proper fit to a Langmuir 1:1 model with high accuracy and reproducibility, thus unraveling subtle differences within RBD mutants and ACE2 glycovariants. Our study emphasizes the importance of SPR-based assay parameters in the acquisition of biologically relevant data and offers a powerful tool to deepen our understanding of the role of the various RBD mutations in ACE2 interaction binding parameters.

## Introduction

The ongoing spread of the coronavirus disease 2019 (COVID-19) caused by severe acute respiratory syndrome coronavirus 2 (SARS-CoV-2) continues to lead to the emergence of new variants and epidemic waves. Several SARS-CoV-2 lineages were defined as variants of concerns (VOCs) by the World Health Organization (WHO), including the B.1.1.7 (Alpha), the B.1.351 (Beta), the B.1.617.2 (Delta) and the B.1.1.529 (Omicron), while the variants B.1.617.1 (Kappa) and B.1.1.7 + L452R (named herein AlphaP) were defined as variants of interest (VOIs)^1^. These variants present key mutations within their spike protein and more specifically in its receptor binding domain (RBD). The RBD interacts directly with the human angiotensin-converting enzyme 2 (ACE2), which is expressed at the cell surface of many cell types and initiates the virus cell entry process^2^. Intensive efforts have thus focused on understanding the binding mechanism of the RBD region to ACE2^3–7^. While these studies have provided key information, affinity and kinetic estimates of the RBD / ACE2 interaction that were reported vary by 10 to 20 folds from one study to the other^3,7–12^. Part of the differences can be attributed to the design of the reported (SPR-based) assays where the surface-immobilized protein, the immobilization strategy and the experimental temperature can greatly influence the derived apparent kinetic and thermodynamic parameters^13,14^.

We here report the development of an optimized SPR-based assay for the kinetic analysis of the interaction between the RBD of seven common variants (Wild type, Alpha, AlphaP, Beta, Delta, Kappa and Omicron) and ACE2. By combining a capture strategy based on coiled-coil interactions with the modulation of the experimental temperature at 10 °C, we were able to reduce common artifacts and record interaction data that were accurately fitted with a Langmuir 1:1 kinetic model. We identified the apparent kinetic constants k_on_ and k_off_ for each RBD/ACE2 interaction. The accuracy of the kinetic fits to a 1:1 model enabled us to evaluate subtle differences in the interaction between ACE2 glycovariants with the wild type RBD. Finally, by adding data points at 25 °C and 37 °C, we were able to perform a preliminary thermodynamic analysis for each RBD/ACE2 to better link our results to mutations and other studies. All in all, our study demonstrates that a proper SPR assay design leads to cleaner data which can be properly explained by a simple model, simplifying interpretation of the results.

## Results

The goal of this study was to develop an optimized SPR-based assay to analyze the interactions between the RBD of important variants with ACE2. For assay validation, we chose the variants of concerns (VOCs) Alpha, Beta, Delta and Omicron as well as two former variants of interest (VOIs) Kappa and AlphaP. All the mutants were compared to the original strain identified in Wuhan, China and referred here as wild type. Table 1 summarizes the mutations in the RBD domain of the variants included in this study.

**Table 1:** RBD variants and their mutations from the original strain (Wuhan).

Wild type (Wuhan)	Alpha B.1.1.7	AlphaP B.1.1.7 + L452R	Beta B.1.351	Delta B.1.617.2	Kappa B.1.617.1	Omicron B.1.1.529
G339						D339
S371						L371
S373						P373
S375						F375
K417			N417			N417
N440						K440
G446						S446
L452		R452		R452	R452	
S477						N477
T478				K478		T478
E484			K484		Q484	A484
Q493						R493
G496						S496
Q498						R498
N501	Y501	Y501	Y501			Y501
Y505						H505

**Figure 1:**
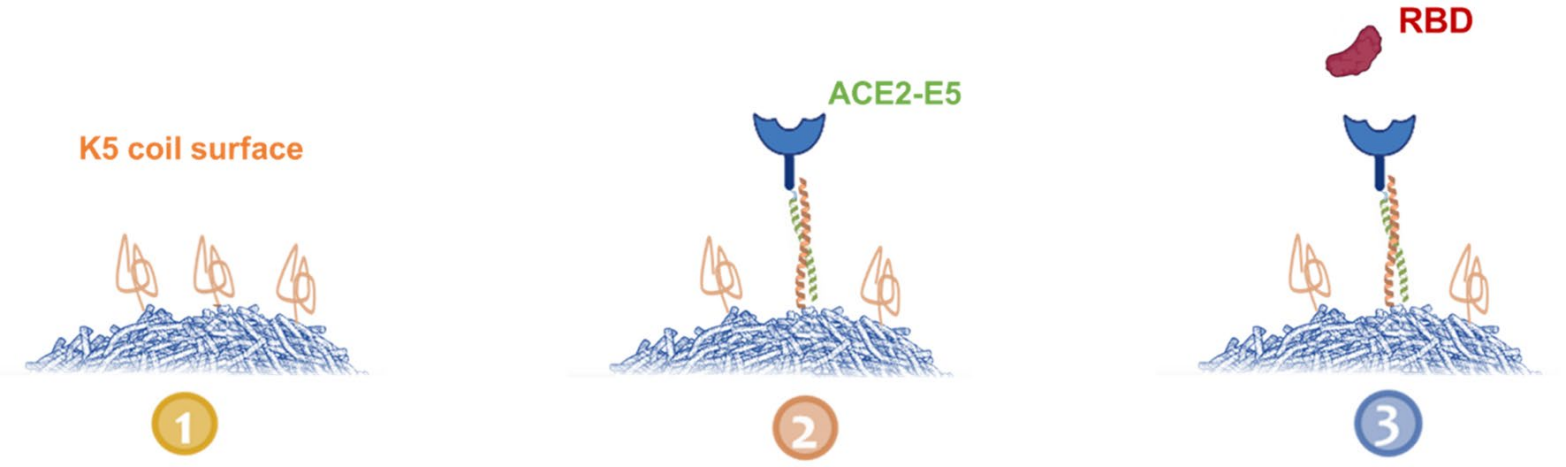
Representation of our three-step SPR-based assay with capture of E5 coil tagged ACE2 (ACE2-E5) by a K5 coil surface.

Our experimental approach relied on two distinct 5 heptad-long (i.e., 35 amino acid-long) non-natural coil peptides denoted E5 and K5 peptides. The E5 coil peptide (EVSALEK heptad) and the K5 coil peptide (KVSALKE heptad) are known to heterodimerize and form a very stable coiled-coil motif through hydrophobic and electrostatic interactions^15,16^. Our previous research work related to monoclonal antibodies binding to Fcγ receptor ectodomains has already demonstrated the various advantages of a coiled-coil based ligand capture approach for SPR-based assays in terms of simplicity, reproducibility, and stability over time. Moreover, compared to other common ligand capture strategies such as anti-His antibodies or streptavidine-biotin, our K5/E5 coiled-coil approach has been shown to reduce artifacts^17^. Here, we covalently immobilized the K5 coil peptides onto the surface of the biosensor via a single engineered cysteine and produced the soluble monomeric ACE2 receptor with a C-terminal E5 coil tag (ACE2-E5) in mammalian cells (Fig. 1).

The E5 coil tag enabled a stable and oriented capture of ACE2 onto the K5 coil functionalized SPR surface. To limit SPR artifacts, which can be caused for example by mass transport limitations, we captured a consistently low amount of ACE2-E5 (~ 60 RUs) leading to a constant maximal resonance signal (R_max_) for all injected RBD variants of 16 ± 3 RUs and we performed every injection cycle at a flowrate of 50 µL/min. A regeneration step (6 M guanidium chloride) promoting the dissociation of the coiled-coil complex was performed to enable the capture of fresh ACE2-E5 for each new cycle. Injecting fresh ligand before each cycle greatly enhances the durability of the surface.

**Figure 2:**
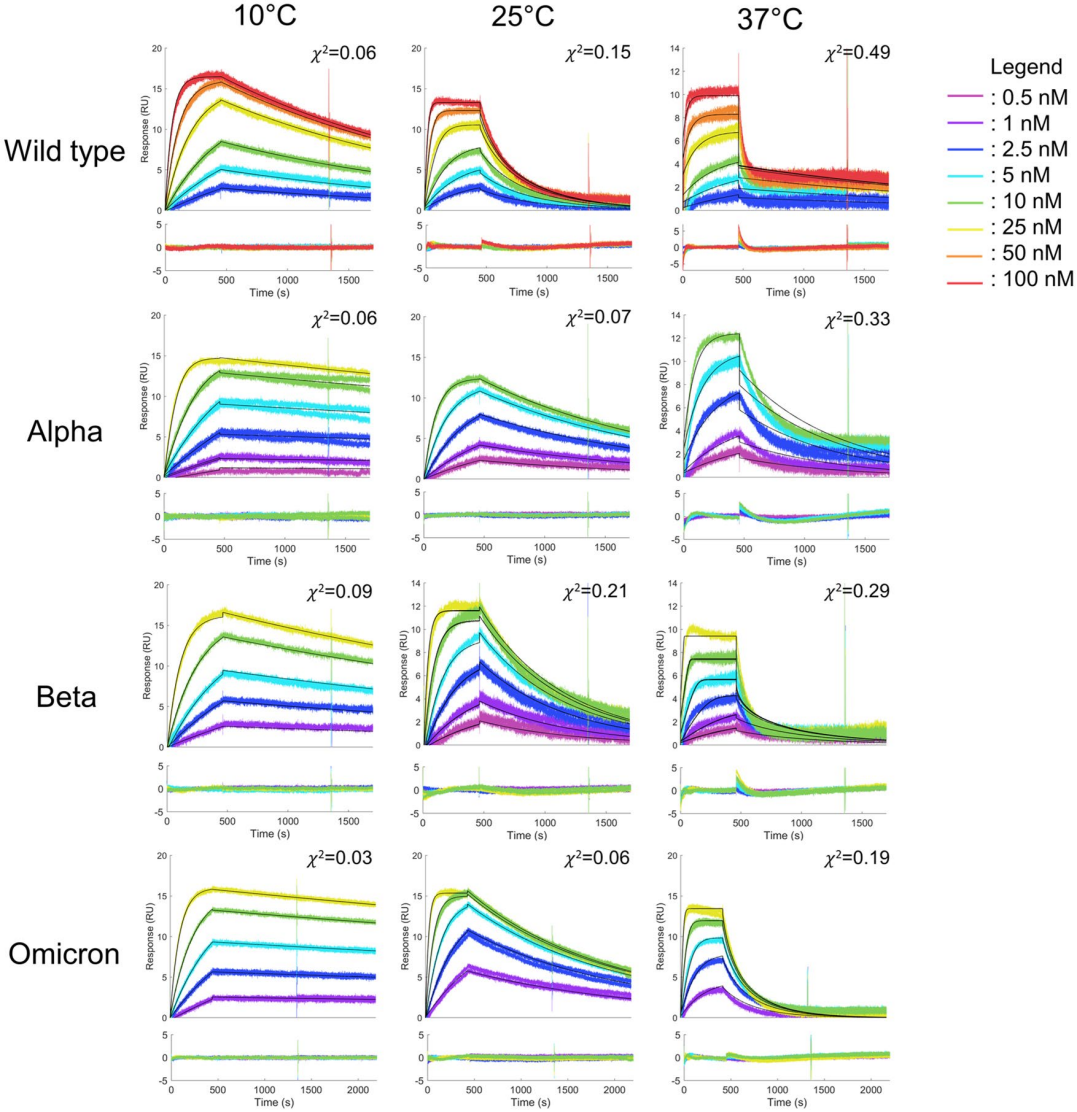
SPR sensorgrams recorded for the RBD variants that do not contain the L452R mutation interacting with tethered ACE2 at different temperatures. The sensorgrams corresponding to variant injections at 10, 25 and 37 °C were globally fitted with a 1:1 Langmuir binding model (solid black lines). Residual plots are included underneath each sensorgram series.

**Figure 3:**
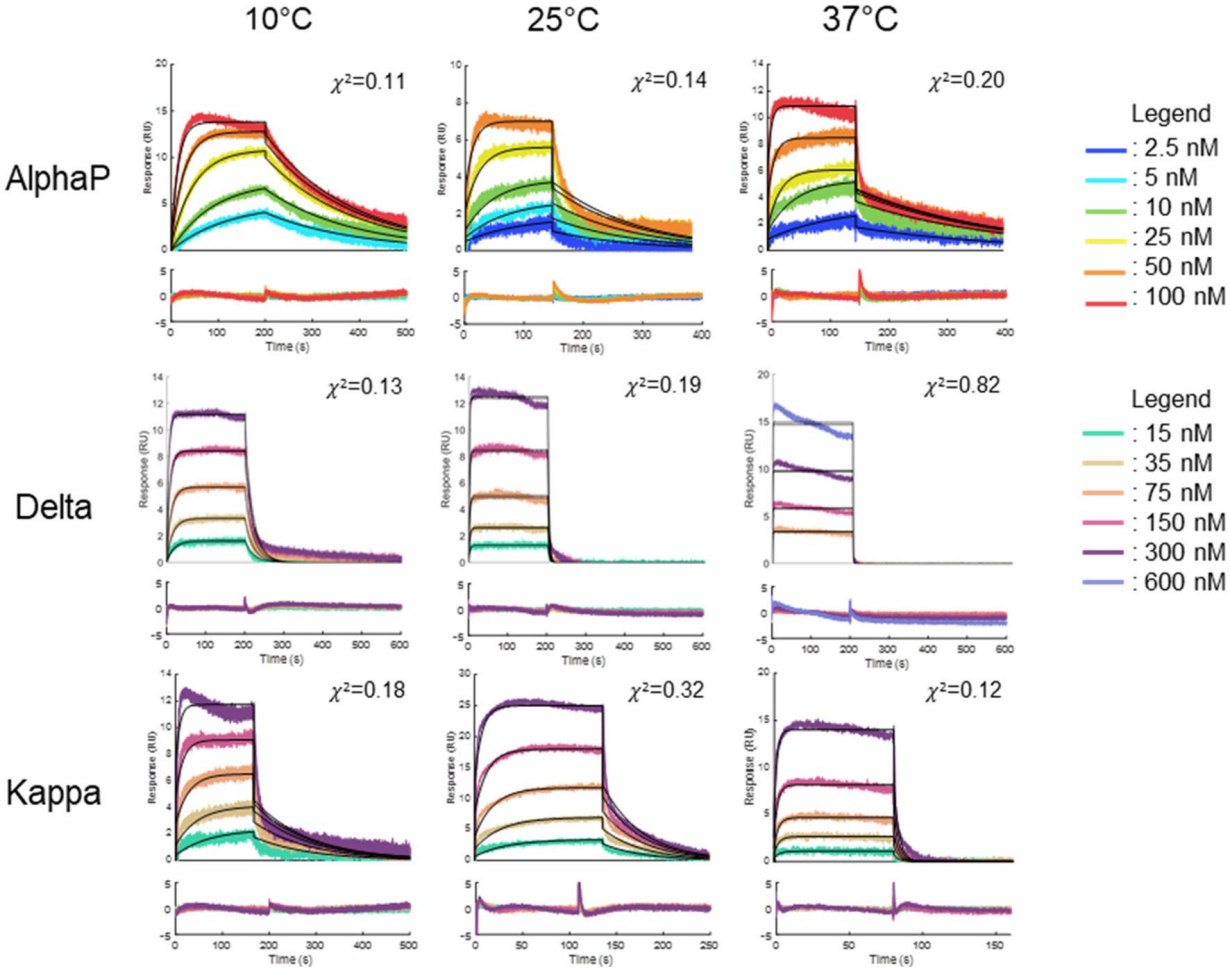
SPR sensorgrams recorded for the RBD variants with the L452R mutation interacting with tethered ACE2 at different temperatures. The sensorgrams corresponding to variant injections at 10, 25 and 37 °C were globally fitted with a 1:1 Langmuir binding model (black solid lines). Residual plots are included underneath each sensorgram series.

We performed all kinetic experiments at three different temperatures (10 °C, 25 °C and 37 °C) and analyzed the recorded sets of sensorgrams with a 1:1 Langmuir interaction model (Figs. 2, 3). We observed clear differences in the sensorgram profiles of the variants that do not contain the L452R mutation (Fig. 2) from the ones that do (Fig. 3). Our data highly suggest that the presence of the L452R mutation accelerates both the association and the dissociation with ACE2.

At 10 °C, we observed excellent fits of the experimental data with a simple kinetic model for all variants. We observed no obvious trend in the residual plots for most sensorgrams recorded at 10 °C and χ^2^ values in the order of 10^–2^ were calculated for all fits to a 1:1 kinetic model at this temperature, which is consequent with the level of measurement noise of the biosensor. Distinct curvatures and spikes were observed in the residual plots at the beginning of the association and dissociation phases for higher temperatures. The adequacy between the collected data and the Langmuir model decreased as the assay temperature increased. This deviations from a simple behavior cannot be attributed to biases related to the coiled-coil mediated capture since, in the case of the Alpha and Omicron RBD mutants, a good adequation, χ^2^ of 0.07 and 0.06 respectively, was still observed at 25 °C. These results show that the combination of our coiled-coil based tethering approach with the lower experimental temperature led to successful fits to a 1:1 kinetic model which enables us to retrieve kinetic data that can be interpreted and compared between RBD variants.

The kinetic constants for the various interactions shown in Fig. 2 determined at 10 °C and 25 °C are given in Table 2.

**Table 2:** Kinetic constants of the interactions between the RBD variants and ACE2 at 10 °C and 25 °C.

RBD variants	10 °C		25 °C	
	k_on_ (10^5^ M^−1^ s^−1^)	k_off_ (10^–4^ s^−1^)	k_on_ (10^5^ M^−1^ s^−1^)	k_off_ (10^–4^ s^−1^)
Wild type	1.76 ± 0.29	4.45 ± 0.32	8.02 ± 1.10	52.80 ± 5.50
Alpha	4.34 ± 0.50	0.79 ± 0.08	11.10 ± 1.53	6.43 ± 1.14
AlphaP	9.46 ± 4.20*	76.7 ± 30.1*	–	–
Beta	3.92 ± 0.41	2.13 ± 0.14	11.39 ± 1.24	16.61 ± 2.74
Delta	4.21 ± 0.31*	447 ± 88*	–	–
Kappa	1.89 ± 0.98*	52.8 ± 18.3*	–	–
Omicron	4.24 ± 0.02	0.57 ± 0.02	17.90 ± 3.59	6.92 ± 0.39

*Indicates kinetic constants for which fits were judged imperfect.

The apparent kinetic constants for the AlphaP, Delta and Kappa variants are provided for an indicative comparison to the other mutants. However, for these variants, the fits to the 1:1 kinetic model are poor. Thus, the derived kinetic constants must rather be regarded as indicative orders of magnitude. The sensorgrams of the AlphaP, Delta and Kappa variants at 25 °C were only analyzed and validated by a steady-state model.

Equilibrium constants (K_D_) were then calculated by computing the ratio of the kinetic constants (k_off_/k_on_) when the simple kinetic model adequately depicted the experimental data. For all other cases, K_D_ was calculated by a steady-state model using the pseudo-equilibrium responses reached at the end of the RBD injections. The standard entropy and standard enthalpy changes were then estimated by a Van’t Hoff analysis (Table 3).

**Table 3:** Thermodynamic evaluation of the interaction of RBD variants with ACE2.

	10 °C − K_D_ (nM)	25 °C − K_D_ (nM)	37 °C − K_D_ (nM)	ΔG° (kcal mol^−1^)	ΔH° (kcal mol^−1^)	ΔS° (cal K^−1^ mol^−1^)
Wild type	2.56 ± 0,35	6.73 ± 0.03	19.14 ± 1.60	− 10.99	− 12.67	− 5.97
Alpha	0.18 ± 0.03	0.64 ± 0.20	2.70 ± 0.40	− 12.43	− 17.45	− 16.97
AlphaP	11.84 ± 6.29	15.61 ± 0.67	22.98 ± 1.56*	− 10.52	− 4.06	21.27
Beta	0.55 ± 0.06	1.48 ± 0.40	5.92 ± 1.33	− 11.71	− 15.30	− 11.95
Delta	106 ± 17	198 ± 48	595 ± 29*	− 9.00	− 10.91	− 6.21
Kappa	29.47 ± 5.58	69.02 ± 4.86	75.5 ± 11.51*	− 9.56	− 9.56	0.48
Omicron	0.14 ± 0.01	0.40 ± 0.07	1.98 ± 0.17	− 12.43	− 16.97	− 14.58

*Indicates kinetic constants that were calculated using a steady-state model.

A Van’t Hoff plot was constructed for each RBD variant and showed good fits with R^2^ coefficients greater than 0.94 in all cases except the Kappa variant (R^2^ of 0.88) (Supplementary Information Fig. S1), thus validating the inherent hypothesis of temperature independence for ΔH° and ΔS° of this system.

Finally, we extended our assay to the evaluation of binding kinetics between various glycoforms of ACE2-E5 with SARS-CoV-2 RBD wild type. Six glycoforms of ACE2-E5 with or without α2,3 sialylation, α2,6 sialylation or fucosylation were produced using glycoengineered Chinese Hamster Ovary (CHO) cells. The thermodynamic constants of these ACE2 glycoforms at 10 °C are presented in Table 4.

**Table 4:** Affinities of various ACE2 glycoforms for wild type RBD.

	K_D_ (nM)	k_on_ (10^5^ M^−1^ s^−1^)	k_off_ (10^–4^ s^−1^)	Glycoform
WT	2.57 ± 0.09	1.76 ± 0.05	4.55 ± 0.04	+ α2,3 sialylation − α2,6 sialylation + Fucosylation
WT/ST6	2.61 ± 0.12	1.68 ± 0.12	4.38 ± 0.11	+ α2,3 sialylation + α2,6 sialylation + Fucosylation
F15	3.68 ± 0.11	1.30 ± 0.02	4.79 ± 0.10	+ α2,3 sialylation − α2,6 sialylation − Fucosylation
dKO2	2.96 ± 0.01	1.58 ± 0.01	4.66 ± 0.02	− α2,3 sialylation − α2,6 sialylation − Fucosylation
dKO2/ST6	3.47 ± 0.07	1.39 ± 0.05	4.81 ± 0.03	− α2,3 sialylation + α2,6 sialylation − Fucosylation
S9	2.35 ± 0.12	1.94 ± 0.02	4.55 ± 0.27	− α2,3 sialylation − α2,6 sialylation + Fucosylation

+ and − signs indicates the presence or absence of the glycosylation type.

The different ACE2 glycoforms were produced in glycoengineered cell lines with knockout genes as previously reported^18^. While F15 and S9 cell lines produce glycoproteins without core-fucose sugar and α-2,3-linked sialic acid, respectively, dKO2 is a double knock-out cell line lacking both core-fucosylation and α2,3 sialylation. The cell lines WT/ST6 and dKO2/ST6 stably express the human *ST6Gal1* gene and are thus capable of generating glycans with terminal α2,6 sialylation. At 10 °C, no statistical differences were observed between the thermodynamic (Table 4) and kinetic constants depicting the interactions of the various ACE2 glycovariants with the wild type RBD.

## Discussion

As part of the global effort towards a better understanding of the infection mechanisms of SARS-CoV-2, we have reported here a surface plasmon resonance (SPR)-based approach allowing for the kinetic evaluation of ACE2 binding to various SARS-CoV-2 RBDs. Different approaches can result in a wide range of thermodynamic and kinetic constants as reported in the case of SARS-CoV-2 variants in interaction with ACE2 (K_D_ varying from 4 to 80 nM)^3,7–12^. Our experimental approach allowed us to depict the data with a 1:1 Langmuir kinetic model and thus draw reliable and meaningful conclusions about the impact of key RBD mutations. SPR-based assays are sensitive to several parameters such as the ligand capture strategy, the ligand density, the flowrate, and the experimental temperature^19^. The experimental conditions must be optimized to minimize potential artifacts, such as mass transport limitations, avidity, steric hindrance, and rebinding, and to fit to a kinetic model representative of the biological interaction^20^. A poor fit to a simple model, or even a good fit to a complex model that does not accurately depict the actual interaction scheme can obfuscate the interpretation or the comparison of the results.

To facilitate the interpretation of the results, our strategy was to focus only on the RBD region instead of using the complete spike protein ectodomain since its trimeric nature may introduce potential artifacts in the kinetic analysis^12,21^. The spike protein is a large trimeric glycoprotein (~ 550 kDa) bearing three RBDs all capable of interacting with an ACE2 receptor. This complex, spike/ACE2, is thus prone to avidity and rebinding artifacts in SPR-based assays. Moreover, it has been shown that each RBD can individually be either in an up or down conformation which influences the overall spike affinity for ACE2 and adds a heterogeneity bias in the analytes of the SPR-based assay^20^. The proportion of RBD in each conformation can vary for different spike variants leading to even more complexity in the analysis of the spike/ACE2 interaction^21,22^. Conducting SPR-based experiments with the RBD as the analyte limits these biases and simplifies the kinetic profiles of the collected data, as the RBD/ACE2 follows, in theory, a 1:1 binding model as we were able to observe at 10 °C. It is then easier to obtain good quality and robust data to better understand the subtle affinity and kinetic differences between SARS-CoV-2 variants and the ACE2 receptor.

Also, we optimized our SPR-based assay by tethering ACE2 onto the biosensor surface in a highly stable and oriented manner. The oriented immobilization of the ligand by a capture agent (such as the coiled-coil peptide system) enables more control and flexibility over the density of ligand on the surface and guaranties the structural integrity of the ligand, on top of reducing heterogeneity in the ligand bioactivity^20,23^. Our coiled-coil mediated strategy allowed us to homogeneously capture a constant low density of fresh ACE2-E5 at the surface for each experimental cycle. A low ligand density reduces the risk of mass transport limitations and rebinding artifacts, which affect the kinetic interpretation of the data^24,25^. Moreover, the coiled-coil system proved to be stable and highly reproducible as we were able to obtain robust and reproducible results over five different sensor chips. The wild type (n = 5) and Alpha variants (n = 6) were our controls for every new sensor chip and showed minimal standard deviations on the calculated constant of affinity K_D_ at 10 °C (2.56 ± 0.35 nM for wild type and 0.18 ± 0.03 nM for Alpha).

We took advantage of our assay to look at the impact of ACE2 glycosylation profiles upon RBD binding, as both ACE2 and RBD/spike are glycoproteins and it has been suggested that RBD and ACE2 glycans may contribute to binding stabilization^26–28^. At 10 °C, we observed slight differences, i.e., less than 1.5-fold, between afucosylated ACE2 glycoforms, (F15, dKO2 and dKO2/ST6) and the WT ACE2 produced in CHO cells. To the best of our knowledge, no other studies observed an influence from fucosylation. Our results did not show a detectable influence from sialylation on the affinity between ACE2 and the wild type RBD. Other studies report a decreased affinity between sialylated ACE2 glycoforms with SARS-CoV-2 SPIKE protein^28–30^. Therefore, increasing the sialylation level of ACE2 might lead to observable differences using our enhanced low-temperature SPR assay. Moreover, it would be interesting to study the glycosylation profile of RBD variants and tests if some have more affinity with particular ACE2 glycovariants. Finally, the next step with our optimized SPR assay, would be to go to the next level of complexity and study the impact of ACE2 N-glycosylation on the complete S1 domain of the spike protein which presents 13 N-glycans, while the RBD region only harbors two N-glycosylation and up to 5 O-glycosylation sites^31^.

Finally, we varied the experimental temperature which showed the importance of this parameter on the measurements of simple kinetics for the RBD/ACE2 interactions. At 37 °C, the interaction kinetics were fast and deviated from a 1:1 kinetic model. This complexity can be attributed to an enhanced mobility and conformation change of the RBD loop interacting with ACE2 at higher temperatures^21,32,33^. Other research groups performed their analysis at 25 °C, which is the most common temperature in SPR-based assays^9,34^. We were able to obtain acceptable fits with a 1:1 Langmuir binding model at 25 °C but slowing the kinetics at an even lower temperature, i.e. 10 °C, enabled us to observe excellent fits and enhanced the differences between the variants, better highlighting the impact of RBD mutations on ACE2 binding.

We report comparable association rate constants between the Alpha, Beta and Omicron variants resulting in ~ 2.5 to 3-time faster binding than the wild type RBD (k_on_ = 1.8 ± 0.3 × 10^5^ M^−1^ s^−1^, Table 2). The differences were more apparent in the dissociation rates where the Omicron and the Alpha presented the slowest rates at 0.57 ± 0.02 × 10^–4^ s^−1^ and 0.79 ± 0.08 × 10^–4^ s^−1^ respectively, which is 5 to 8 times slower than the wild type RBD. Meanwhile, the dissociation rate of the Beta variant is only 2 times slower. These kinetics confer the Omicron and the Alpha RBD variants with the highest affinities for ACE2, with ~ 20- and 15-fold decreases in K_D_ compared to the wild type RBD, respectively. The increased affinity of the Alpha variant is linked to the only RBD mutation, N501Y, more likely due to the addition of a π-stacking interactions with the Y41 side chain of ACE2^26,35,58^. The observed binding enhancement for the Omicron variant, however, is more complex to dissect due to the high number of mutations present at the interface with ACE2. Recent in silico models highly suggest that four key mutations contribute to this stronger interaction: S477N, G496S, Q498R and N501Y^36^. Other point mutations, such as H505Y and K417N, were observed to negatively impact the interactions with ACE2, which modulates the affinity to a similar level to that of the Alpha variant^37–39^. Further work is still needed to understand the individual and/or combine influence of Omicron mutations on its affinity for ACE2.

**Figure 4:**
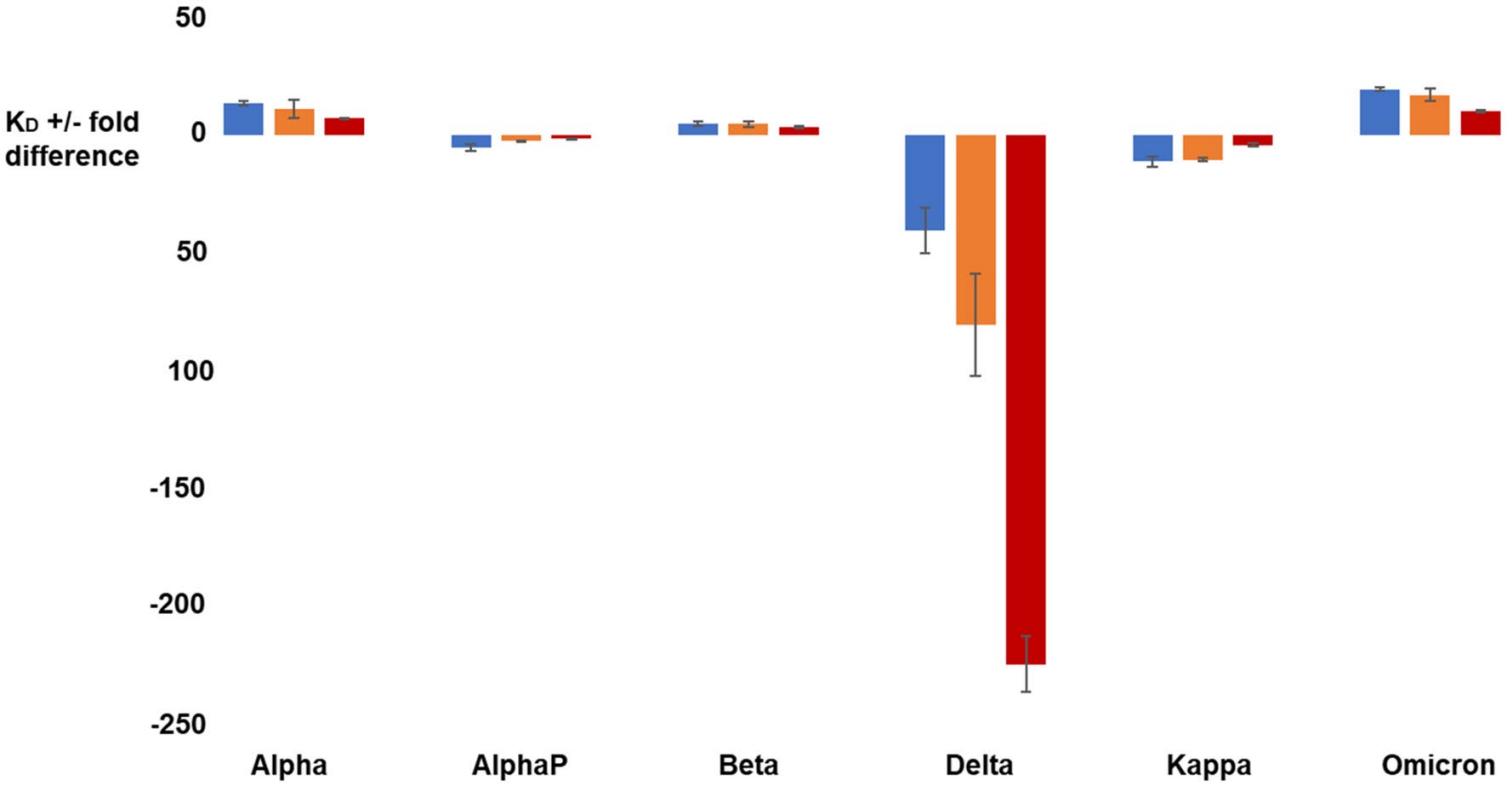
Comparison of the affinity of the RBD/ACE2 interaction at 10 °C (in blue), 25 °C (in orange) and 37 °C (in red) for all RBD variants considered in this study. The fold difference with respect to the affinity of the wild type RBD at a given temperature is reported.

Only a five-fold affinity enhancement compared to the wild type at 10 °C is observed for the Beta-ACE2 complex, where the positive effect of the N501Y mutation seems to be counteracted by the loss of a salt bridge by RBD K417N mutation with ACE2 D30^40^. The absence of this salt bridge translates to a decreased affinity as observed in this work (Fig. 4) and in several other studies^7,34,41^. We found no significant impact of the E484K mutation on the binding affinity to ACE2, while other studies have reported conflicting results^9,36,42^.

The AlphaP (L452R/N501Y), Delta (L452R/T478K) and Kappa (L452R/E484Q) variants all showed faster association and dissociation rates at all temperatures. They also exhibited a lower affinity for ACE2, i.e., 5-, 40- and ten-fold decreases respectively compared to the wild type, at 10 °C. Based on these results, we could infer that the L452R mutation disturbs the binding to ACE2 and that, while it is compensated by the N501Y mutation in AlphaP, its effect is more apparent in Delta and Kappa. These results contrast with some studies that measured similar or slightly higher affinities for these variants compared to wild type^43,44^. However, the impact of L452R mutation on the RBD / ACE2 interaction is still debated as several studies demonstrated that it does not play a major role in the interaction with ACE2^5,45,46^. In opposition, some have suggested that the L452R mutation abrogates a hydrophobic patch formed by the amino acids L452, L492 and F490^47^. The loss of this patch could impact stability of the RBD and possibly its complexation with ACE2, as suggested by our results. Moreover, both mutations L452R and E484Q are not involved in the interaction with ACE2 and most studies have linked them to an enhanced immune evasion from neutralizing antibodies^40,48–51^. However, the T478K mutation on Delta has been linked to a higher affinity for ACE2 due to the introduction of a positive charge on the lysine residue^52^. Some studies have shown, in the context of a trimeric spike construct, that the apparition of a positive electrostatic charge from T478K mutation stabilizes the RBD and favors its up conformation, leading to a better interaction with ACE2^32,33,44^.

Thermodynamic differences were also apparent in the enthalpic and entropic values measured by Van’t Hoff plots for the AlphaP and Kappa. Enthalpic changes favor binding in all cases, as is inferred from a negative ΔH°, indicative of an exothermic reaction. Amongst the variants that were studied, Alpha, Beta and Omicron showed the most exothermic behavior, whereas enthalpic contributions were the weakest for Kappa and AlphaP. Entropic contributions showed the opposite trend. They were shown to hamper binding to ACE2 for Alpha, Beta, Delta and Omicron (and wild type) whereas they contributed to the stability of the RBD/ACE2 complex for Kappa and AlphaP. The L452R mutation could be in part responsible for the opposite trends in enthalpic and entropic changes, as it is common to both Kappa and AlphaP. The L452R impact on Delta RBD/ACE2 thermodynamic values could be balanced by the T478K mutation which integrates a second positive charge and was shown to contribute to RBD conformation stabilization^33,49^.

In summary, we present an optimized SPR-based assay to enhance our understanding of the binding mechanisms between SARS-CoV-2 RBD and the human ACE2. The use of the K5/E5 coiled-coil tethering strategy resulted in a high reproducibility in our results while a low experimental temperature of 10 °C enabled us to collect data depicted by a simple 1:1 Langmuir kinetic model. Altogether, this allowed us to precisely characterize the interactions of SARS-CoV-2 RBD variants with ACE2 and compare them with each other. This approach could be used to rapidly compare new variants of concern to those reported here and infer on their capacity to infect human cells.

## Materials and methods

### Plasmids

The human ACE2 cDNA (UniProtKB-Q9BYF1) was synthesized by GenScript and optimized for expression in CHO cells. The construct encodes a human interleukin 10 signal peptide (MHSSALLCCLVLLTGVRA) followed by a Twin-Strep-tag II-(His)_6_-FLAG tag on the N-terminus of the mature ACE2 receptor ectodomain (amino acids 20–613). An E5 coil sequence flanked by glycine linkers (GGGG[EVSALEK]_5_GGG) was added in-frame at the ACE2 C-terminus. The cDNA was cloned into pTT5® expression using EcoRI and BamHI restriction enzymes.

Human beta-galactoside alpha-2,6-sialyltransferase 1 (*ST6Gal*1 or hST6) was also cloned into pTT® vector as described previously to modify the glycosylation of the ACE2 receptor^53^.

The RBD sequences (RBD^319–541^) of the SARS-CoV-2 spike protein (YP_009724390.1, Wuhan-Hu-1 strain or its variants) encodes a C-terminal (His)_6_-FLAG tag and were cloned into the pTT5® vector using EcoRI and BamHI.

### Protein expression and cell culture

*Fut8* (F15), *ST4Gal4* (S9) as well as *Fut8* and *ST3Gal4* (dKO2) knock-out CHO cell lines were derived from CHO^55E1^ cells^54^ using CRISPR/Cas9 as described elsewhere^18^ and were used for transient ACE2 receptor expression following a previously reported protocol^55,56^. In brief, cells were maintained in a chemically-defined proprietary media formulation supplemented with 4 mM l-glutamine and incubated in shake flasks (Corning, NY, USA) under agitation (120 rpm) at 37 °C, 5% CO_2_. Two days prior to transfection, cells were seeded at 1 × 10^6^ cells/mL in the same media to achieve a cell density of ∼8 × 10^6^/mL on the day of transfection. Right before transfection, cells were diluted with 25% fresh media and dimethylacetamide was added to 0.083% (v/v). PEI-Max (Polysciences) was used to transfect cells at a DNA:PEI (polyethylenimine) ratio of 1:7 (w:w) and plasmid DNA final concentration was 1.4 μg/mL in cell culture media. The transfected DNA was a mix of 85% (w/w) of the different pTT5-RBD constructs or pTT241-ACE2 ECD, 10% pTT-Bcl-XL (anti-apoptotic effector) and 5% pTT-GFP. The hST6 expression vector was co-transfected with ACE2-ECD vector in dKO2 cells (dKO2/ST6) and WT CHO cells (WT/ST6). At 24 h post-transfection, expression of the ACE2 was induced by adding 2 μg/mL of cumate and all cultures were supplemented with Anti-Clumping Supplement (1:500 dilution) (Irvine Scientific) as well as Feed 4 (2.5% v/v) (Irvine Scientific) before moving to a 32 °C incubator. At 5 days post-transfection, cultures were fed with additional 5% of Feed 4 and additional glucose was added every 2–3 days to maintain a minimal concentration of 17 mM. Cell supernatants were collected at 6–7 days post-transfection.

### Protein purification

All constructs were purified essentially as previously described^31^. A first immobilized affinity chromatography (IMAC) step was performed using Nickel Sepharose Excel resin (GE Healthcare). Columns were equilibrated with equilibration buffer (50 mM NaPO_4_ pH 7.8, 300 mM NaCl) and supernatants were loaded at 3 mL/min. Columns were washed once with 50 mM NaPO_4_ pH 7.8, containing 300 mM NaCl and 10 mM imidazole and eluted with the same buffer containing 300 mM imidazole. Wild type RBD and its variants were further purified using Superdex75 gel filtration column (GE Healthcare) except for the Omicron variant that was further purified by anti-FLAG chromatography^55^ prior to Superdex75 gel filtration. This is necessary to separate monomers from dimers. The IMAC-purified ACE2 protein was loaded on Strep-Tactin XT Superflow high-capacity resin (IBA Lifesciences, Germany) according to the manufacturer’s instructions. Buffer exchange was done with desalting columns (GE Healthcare) with PBS. Purified proteins were quantified using a NanoDrop Spectrophotometer (ThermoFisher). Finally, SDS-PAGE and total protein staining (Coomassie Blue) were performed using standard methods.

### Surface plasmon resonance (SPR)

SPR experiments were performed using a Biacore T100 system (GE Healthcare) and research-grade CM5 sensor chips from Cytiva (Series S Sensor chip CM5, cat #29104988). The running buffer was HBS-EP + (10 mM HEPES, 0.15 M NaCl, 3 mM ethylenediaminetetraacetic acid (EDTA), and 0,05% [v/v] surfactant P20, pH 7,4) from Cytiva as well (cat #BR100669).

Cysteine-tagged K5 peptides (CGG-[KVSALKE]_5_) were synthesized by the peptide facility at University of Colorado, as previously described^57^. K5 peptides were covalently immobilized on the carboxymethyl-dextran sensor surface at about 1200 RUs on the reference and experiment flow cells as described by Murschel et al.^15^. E5 coil tagged ACE2 receptors, diluted at 1 μg/mL, were captured on the experiment surface previously functionalized with the K5 peptide, via E5/K5 coiled-coil interactions at a flow rate of 10 μL/min. Approximately 60 RUs of ACE2 receptors were captured on the surface for all experimental cycles, for all RBD variants and temperatures. No ACE2 was injected over the reference surface. The surfaces were regenerated with three 15 s pulses of 6 M guanidium/HCl at 100 μL/min.

The Wild type, Alpha, Beta and Omicron RBD variants were injected for 460 s over the captured ACE2 receptors and control surfaces at a flow rate of 50 μL/min at six concentrations between 0.5 nM and 100 nM in duplicate or triplicate. The dissociation was then monitored for 1250 s for Wild type, Alpha and Beta, and 1800s for Omicron. As for the AlphaP and Kappa RBD variants, the injection was conducted between 80 and 200 s depending on the experimental temperature and the dissociation was monitored for at least 150 s. The experimental temperatures were 10 °C, 25 °C and 37 °C. Each experiment was repeated at least 2 times and up to 6 times at 10 °C. The data acquisition frequency was set to 10 Hz.

Sensorgrams were referenced by subtracting the signal recorded on the reference surface (with no ACE2) to that recorded on the active surface (with ACE2). Blank injections were also performed to subtract out the effects of buffer changes, temperature drifts and mechanical artifacts. This process is called double-referencing^13^.

### Data analysis

The interaction between the immobilized ACE2 and the injected RBD (and variants) was assumed to follow a 1:1 binding model. As sensorgrams corresponding to multiple RBD concentrations were recorded, global fitting was performed for each temperature and variant. Both kinetic (k_on_ and k_off_) and thermodynamic (K_D_) constants were identified. The fits were performed via the Biacore Evaluation Software. To evaluate the appropriateness of the one-to-one binding assumption, a residual analysis was performed by comparing the recorded sensorgrams to the predicted sensorgrams calculated with the identified parameters. The standard deviation of the parameters obtained at different repetitions of the experiments were calculated to assess the repeatability of the SPR assay.

Van’t Hoff plots were used to determine the standard reaction enthalpy and entropy via their slope and intercept. They are based on the Van’t Hoff equation:

$${R}_{IG}T\mathrm{ln}\left({K}_{D}\right)=\Delta G^\circ =\Delta H^\circ -T\Delta S^\circ$$

1 $$\mathrm{ln}\left({K}_{D}\right)=\frac{\Delta H^\circ }{{R}_{IG}T}-\frac{\Delta S^\circ }{{R}_{IG}}$$

${R}_{IG}$ denotes the ideal gas constant and $T$ is the temperature. $\Delta G^\circ$ is the Gibbs free energy of the pseudo-reaction, while $\Delta H^\circ$ and $\Delta S^\circ$ are the standard reaction enthalpy and the standard reaction entropy, respectively.

## Supplementary Information

Supplementary Figure S1.

## Data Availability

The datasets generated during and/or analysed during the current study are available from the corresponding author on reasonable request.
