# Supplementary figures and images for: Impact of the temperature on the interactions between common variants of the SARS-CoV-2 receptor binding domain and the human ACE2
Source: Sci Rep. 2022 Jul 7;12:11520. doi: 10.1038/s41598-022-15215-5 (PMC9261887; doi:10.1038/s41598-022-15215-5)

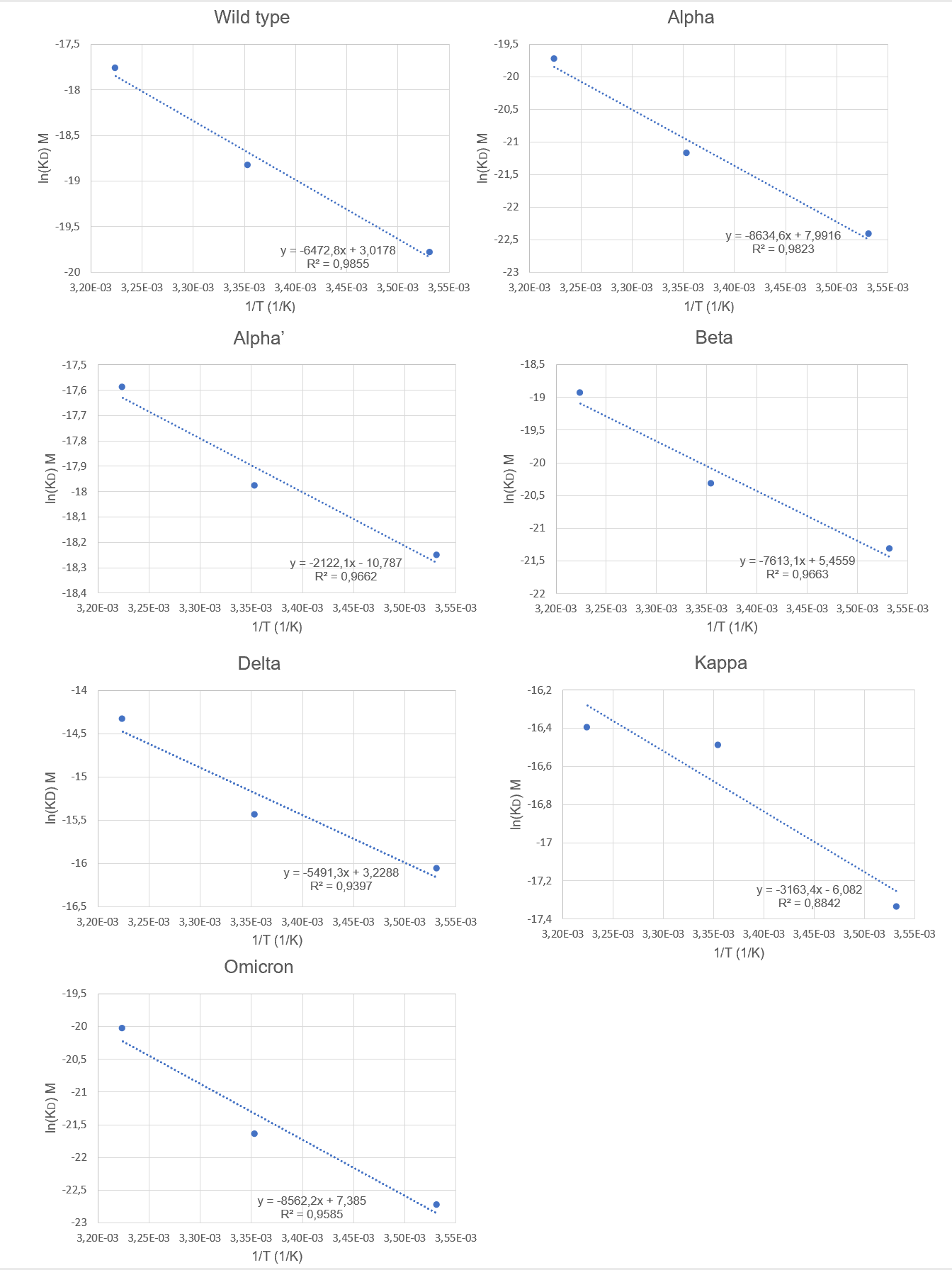


Figure S1 Van't Hoff plot for each RBD variants

Supplement: Supplementary file 1 — Supplementary Figure S1. [file 41598_2022_15215_MOESM1_ESM.docx]
